# Transcriptome analysis of the uterus of hens laying eggs differing in cuticle deposition

**DOI:** 10.1186/s12864-020-06882-7

**Published:** 2020-07-27

**Authors:** Sandra Poyatos Pertiñez, Peter W. Wilson, Wiebke Icken, David Cavero, Maureen M. Bain, Anita C. Jones, Ian C. Dunn

**Affiliations:** 1 The Roslin Institute, University of Edinburgh, Easter Bush Campus, Midlothian, EH25 9RG Scotland, UK; 2 Lohmann Tierzucht, 7454 Cuxhaven, Germany; 3 H&N International, 27472 Cuxhaven, Germany; 4 College of Medical, Veterinary and Life Sciences (MVLS), IBAHCM, University of Glasgow, Glasgow, Scotland, UK; 5 School of Chemistry, University of Edinburgh, Joseph Black Building, Edinburgh, Scotland, UK

**Keywords:** Uterus, Egg, Oviduct, Cuticle, Oviposition, Transcriptome, Next generation sequencing, Chicken

## Abstract

**Background:**

Avian eggs have a proteinaceous cuticle. The quantity of cuticle varies and the deposition of a good cuticle in the uterus (Shell-gland) prevents transmission of bacteria to the egg contents.

**Results:**

To understand cuticle deposition, uterus transcriptomes were compared between hens with i) naturally good and poor cuticle and, ii) where manipulation of the hypothalamo-pituitary-gonadal-oviduct axis produced eggs with or without cuticle. The highest expressed genes encoded eggshell matrix and cuticle proteins, e.g. MEPE (OC-116), BPIFB3 (OVX-36), RARRES1 (OVX-32), WAP (OVX-25), and genes for mitochondrial oxidative phosphorylation, active transport and energy metabolism. Expression of a number of these genes differed between hens laying eggs with or without cuticle. There was also a high expression of clock genes. *PER2, CRY2, CRY1, CLOCK* and *BMAL1* were differentially expressed when cuticle deposition was prevented, and they also changed throughout the egg formation cycle. This suggests an endogenous clock in the uterus may be a component of cuticle deposition control. Cuticle proteins are glycosylated and glycosaminoglycan binding genes had a lower expression when cuticle proteins were deposited on the egg. The immediate early genes, *JUN* and *FOS*, were expressed less when the cuticle had not been deposited and changed over the egg formation cycle, suggesting they are important in oviposition and cuticle deposition. The uterus transcriptome of hens with good and poor cuticle deposition did not differ.

**Conclusions:**

We have gained insights into the factors that can affect the production of the cuticle especially clock genes and immediate early genes. We have demonstrated that these genes change their expression over the period of eggshell formation supporting their importance. The lack of differences in expression between the uterus of hens laying eggs with the best and worse cuticle suggest the genetic basis of the trait may lie outside the oviduct.

## Background

The cuticle that covers the eggs of many bird species is invisible [1]. Perhaps for that reason it has been a relatively neglected structure. However, invisibility does not mean it is not important, for birds that live in challenging aquatic environments and where the nests are dirty it is of critical importance for the prevention of bacterial contamination and tends to be much thicker the greater the challenge [1–3]. Conversely, species from drier environments seem to have less cuticle or cuticle associated structures [4, 5]. The cuticle is a highly glycosylated proteinaceous layer that is specifically deposited on the outside of avian eggs [6], filling the pores in the shell, so preventing aqueous access and bacterial contamination but still allowing gas exchange [7–9]. The predominant cuticle proteins in the chicken appear to be the BPI fold containing family B member 3 (BPIFB3 also known as ovocalyxin-36), kunitz-like protease inhibitor, matrix extracellular phosphoglycoprotein (MEPE also known as ovocleidin-116)*,* ovocleidin-17 (OC-17), ovocalyxin 25 (WAP), clusterin (CLU) and retinoic acid receptor responder 1 (RARRES1 also known as ovocalyxin-32) [10]. Lower levels of other proteins have been reported depending on the method used to recover the cuticle [11–13]. Despite these proteins having a not dissimilar constitution to the organic matrix of the shell [14], termination of shell formation occurs before the deposition of the cuticle [6]. Therefore, cuticle deposition is not an extension of shell organic matrix production but is a specific event that occurs in the uterus, often referred to as the shell gland pouch, very close to the time of oviposition [6]. This was elucidated by utilizing precise temporal manipulation of the hypothalamo-pituitary-gonadal-oviduct axis to manipulate the daily laying cycle and obtain eggs at the same time in the daily cycle which had cuticle deposition or did not have cuticle deposition [6]. However, from this study it became obvious that secretion of the components of the cuticle are not controlled by the proximate endocrine and paracrine factors responsible for the act of oviposition, namely arginine vasotocin (AVT) and prostaglandin [6]. The question of what precise pathways are responsible for the secretion of the cuticle however, remains unresolved.

Previously we speculated that there may even be intrinsic timing events that control the succession of events in the uterus from mineralization through to cuticle deposition and oviposition [6]. We might also expect that factors which are responsible for the overall maintenance of a fully developed oviduct and which act to prevent apoptosis would be important. These factors would affect the overall quality of the cuticle and the expression of genes which control this, these factors might include the receptors for ovarian steroids [15] and growth factors thought to interact with them [16, 17]. We also demonstrated on multiple lines of divergent breeds of chicken that the quantity of cuticle is a heritable trait and the hen’s level of cuticle deposition on eggs was remarkably constant through its life [10, 18]. Most importantly, the quantity of cuticle had a significant effect on bacterial penetration of eggs [8, 10, 19]. It seems therefore, that the vertical and horizontal transmission of bacteria that threaten the developing embryo [20, 21] are prevented by the deposition of a good cuticle. However, it is not known what genetic or environmental factors influence the quantity of cuticle deposited.

Given the state of knowledge about the cuticle and its deposition, we undertook the current study to try and elucidate some of these unanswered questions. Namely, what genes or pathways are involved in the deposition of the cuticle and what genes contribute to the variation in cuticle deposition within a chicken breed? By generating uterus transcriptomes from hens that produced eggs with good or poor cuticle coverage and from hens that were manipulated to produce eggs with or without cuticle, our intention was to increase both our understanding of the uterus transcriptome and to find new avenues to pursue in understanding the biology of this fascinating protective coating found on the surface of eggs.

## Results

### Zoo-technical data and cuticle measurement in hens from experiments 1 and 2

#### Experiment 1: comparison of the top and tail of the cuticle deposition distribution

At post mortem, there was no difference in body weight between the hens sampled from the top (High) or the bottom (Low) of the distribution for cuticle quality (1968 ± 142 g v 1925 ± 215 g; *n* = 8; F1,14 0.65) nor the oviduct weight (82.5 ± 6.7 v 81.3 ± 8.8 g; *n* = 8; F1,14 0.76). As it was the basis of selection of the samples, the cuticle (ΔAbs @640 nm) was more abundant in the High group versus the Low group as indicated by a larger difference in absorbance (High 0.374 ± 0.009 v Low 0.153 ± 0.16 ΔAbs @640 nm; *n* = 8; F1,14 < 0.001). However, there was less pigment on the High group eggs as indicated by the lower pre-test absorbance measurement (High 0.201 ± 0.011 v Low 0.286 ± 0.016 Abs @ 640 nm; *n* = 8; F1,14 < 0.001).

#### Experiment 2: manipulation of cuticle deposition by endocrinological intervention

At post mortem, there was no difference in body weight between the hens used for RNA sequencing transcriptome analysis that received AVT or GNRH1 (1812 ± 138 g v 1732 ± 189 g; *n* = 8; F1,14 0.35). Nor was there a difference in the oviposited egg weight (64.6 ± 6.9 v 63.2 ± 5.1; *n* = 8; F1,14 0.66) or the oviduct weight (82.8 ± 8.5 v 75.8 ± 5.9; *n* = 8; F1,14 0.07) although the latter tended to be heavier in the AVT group. Comparing the egg cuticle (ΔAbs @640 nm) in the two treatments there was little or no cuticle in the AVT group compared to the GNRH1 treated group eggs (GNRH 0.18 ± 0.050 v AVT -0.016 ± 0.10 ΔAbs @640 nm; *n* = 8; F1,14 0.01). Pigmentation of the egg (Abs @ 640 nm) was also affected with a lower absorbance in the AVT treated groups eggs compared to the GNRH1 group which equates with a less pigmented egg (GNRH1 0.361 ± 0.009 v AVT + 0.168 ± 0.008 Abs @ 640 nm; *n* = 8; F1,14 < 0.001).

### Analysis of uterus RNA-sequencing data

#### Experiment 1: comparison of the top and tail of the cuticle deposition distribution

Eight biological replicate samples of chicken uterus from each of the extremes of the distribution of cuticle deposition, i.e. High and Low cuticle quality, were analysed. Quality control and data processing steps rejected one replicate from the High cuticle deposition group resulting in 7 High and 8 Low biological replicates of “clean” data totalling 136,267,433 and 185,605,401 aligned reads respectively. Of a total of 18,346 genes present in the chicken genome, our sequencing reads were assembled onto 12,253 genes (RPKM > 1) (Additional file 1: Table S1). To identify the DEGs between the two groups, we used EdgeR with adjusted *P* < 0.05 for all comparisons. No DEGs were detected between the samples from the High and Low groups.

#### Experiment 2: manipulation of cuticle deposition by endocrinological intervention

**Fig. 1:**
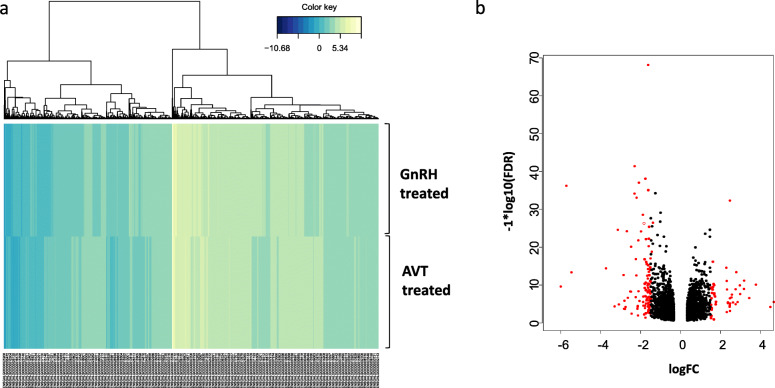
Differential expression of genes extracted from RNA-seq data involved in response to AVT and GnRH1 treatment. **a** Hierarchical clustering analysis of transcription profiles based on 3431 DEGs (yellow, induced genes; blue, repressed genes). **b** Volcano plot showing statistical significance (−log10 FDR) against log-fold change, with DEGs showing a fold-change > 1.5 or < −1.5 highlighted in red

Eight biological replicate samples of chicken uterus in each of two well-studied experimental manipulations of oviposition timing; i.e. the injection of GNRH1 and AVT were available. Quality control and data processing steps rejected one replicate from the AVT treatment group resulting in 8 GNRH1 and 7 AVT biological replicates of “clean” data totalling 129,731,248 and 197,066,236 aligned reads respectively. Of a total of 18,346 genes present in the chicken genome, our sequencing reads were assembled onto 12,248 genes (RPKM > 1) (Additional file 2: Table S2)**.** To identify the DEGs between the two groups, we again used EdgeR with adjusted *P* < 0.05 for all comparisons. A total of 3431 DEGs were detected between the treatment groups (Fig. 1a; Additional file 3: Table S3). Of these, 1736 were significantly up-regulated and 1695 were significantly down-regulated in GNRH1 treated hens compared to AVT hens (Fig. 1b), indicating that uterine genes are differentially expressed in these two treatment groups which because one group deposited cuticle whilst the other did not may be related to this process. Genes from each group with a fold-change > 1.5 or < − 1.5 (46 and 115, respectively) were selected for further analysis (Fig. 1b; Additional file 4: Table S4 and Additional file 5: Table S5**).**

### Expression profile of genes in the uterus close to oviposition in experiment 1 and 2

In the uterus, the 30 genes with the highest expression are detailed in Table 1. There logCPM values are between 11 and 15.3, where the genes in the data set as a whole had a mean expression of 4.4 and an upper quartile value of 6.1 (Table 1).

**Table 1:** Profile of the thirty top expressed genes (log CPM > 11) across experiment 1 and 2

Gene ENSGALG code	Gene description	Gene name	LogCPM Average	logCPM Experiment 1	logCPM Experiment 2
ENSGALG00000010927	matrix extracellular phosphoglycoprotein/Ovocleidin-116	MEPE	15.35	15.55	15.16
ENSGALG00000018373	cytochrome c oxidase subunit I	COX1	15.19	15.13	15.24
ENSGALG00000020972	whey acidic protein	WAP	13.99	14.31	13.66
ENSGALG00000009594	retinoic acid receptor responder 1/ovocalyxin-32	RARRES1	13.97	13.96	13.98
ENSGALG00000018367	cytochrome c oxidase subunit III	COX3	13.79	13.80	13.77
ENSGALG00000018370	cytochrome c oxidase subunit II	COX2	13.42	13.37	13.46
ENSGALG00000006662	BPI fold containing family B member 3/Ovocalyxin-36	BPIFB3	13.13	13.02	13.24
ENSGALG00000018360	Cytochrome b	CYTB	13.07	12.93	13.20
ENSGALG00000018368	ATP synthase membrane subunit 6	ATP6	13.05	13.10	13.01
ENSGALG00000018361	NADH ubiquinone oxidoreductase core subunit 5	ND5	12.59	12.48	12.71
ENSGALG00000015917	Eukaryotic translation elongation factor 1 alpha 1	EEF1A1	12.30	12.38	12.23
ENSGALG00000015003	ATPase Na+/K+ transporting subunit alpha 1	ATP1A1	12.29	12.32	12.25
ENSGALG00000018364	NADH-ubiquinone oxidoreductase chain 4	ND4	12.19	11.96	12.43
ENSGALG00000018382	NADH-ubiquinone oxidoreductase chain 1	ND1	12.19	12.40	11.98
ENSGALG00000018378	NADH-ubiquinone oxidoreductase chain 2	ND2	11.96	11.88	12.04
ENSGALG00000009621	Actin beta	ACTB	11.91	11.92	11.89
ENSGALG00000014442	Glyceraldehyde-3-phosphate dehydrogenase	GAPDH	11.88	12.41	11.34
ENSGALG00000003578	Fibronectin 1	FN1	11.64	11.39	11.88
ENSGALG00000015914	Calbindin 1	CALB	11.55	10.88	12.23
ENSGALG00000028749	actin gamma 1	ACTG1	11.54	11.75	11.33
ENSGALG00000021552	member of RAS oncogene family	RAP2B	11.39	11.88	10.90
ENSGALG00000008684	eukaryotic translation initiation factor 4A2	EIF4A2	11.36	11.23	11.48
ENSGALG00000004509	Polyubiquitin-B	UBB	11.34	11.44	11.25
ENSGALG00000018384	Novel mitochondrial gene		11.33	11.49	11.18
ENSGALG00000021139	immunoglobulin lambda-like polypeptide 1	IGLL1	11.25	12.40	10.09
ENSGALG00000006512	heat shock 70 kDa protein 8	HSPA8	11.11	10.98	11.25
ENSGALG00000004860	dexamethasone-induced 1	RASD1	11.10	11.41	10.78
ENSGALG00000005587	eukaryotic translation initiation factor 4 gamma 2	EIF4G2	11.07	10.88	11.26
ENSGALG00000013257	lactate dehydrogenase B	LDHB	10.97	11.18	10.75

### GO functional annotation and pathway enrichment analysis of the DEGs in experiment 2

DEGs in experiment 2 were enriched in biological process, cellular component, and molecular function categories by GO analysis (http://www.geneontology.org/). GO terms with *P* < 0.05 were considered significantly enriched in DEGs. The GO term enrichment analysis showed that there were no enriched categories of GO functional annotation or pathway enrichment for up-regulated genes in GNRH1 treated hens compared to AVT hens with a fold change ≥1.5 when subject to analysis by the DAVID Functional Annotation Tool.

The top 10 significant GO functional annotation terms for down-regulated genes in GNRH1 treated hens compared to AVT hens are listed in Table 2. In the Biological process ontology, the highest enrichment of DEGs included signal transduction (26 genes), system development (19 genes) and cell differentiation (19 genes). In the Molecular function ontology, only the glycosaminoglycan binding category (GO:0005539) satisfied the cut-off criteria (*P* < 0.05) and included 6 genes (*HBEGF, CYR61, THBS1, CEMIP, SPP1, REG4*). There were no over-represented categories in the cellular component ontology.

**Table 2:** The enrichment of DEGs in GO terms for down-regulated genes in GNRH1 treated hens compared to AVT hens

Category	GO ID and term	Count	*P*-value	Genes
Biological process ontology	0043618: regulation of transcription from RNA polymerase II promoter in response to stress	3	1.30E-04	*KLF2, JUN, ATF3*
	0070372: regulation of ERK1 and ERK2 cascade	6	8.16E-06	*CYR61, CCAH221, SLC30A10, SPRY2, JUN, ATF3*
	0006469: negative regulation of protein kinase activity	5	2.00E-04	*SOCS1, DUSP8, DUSP6, SPRY2, ERRFI2*
	0009888: tissue development	13	2.57E-06	*HBEGF, NEXN, TXK, CYR61, TIPARP, FOS, SPRY2, KLF2, JUN, ATF3, ARC, NR4A3, ERRFI2*
	1901700: response to oxygen-containing compound	9	1.07E-04	*KCNJ11, SOCS1, FOS, TNFRSF6B, SLC30A10, HSP70, JUN, NR4A3, PDK4*
	0009605: response to external stimulus	13	3.47E-06	*HBEGF, CH25H, KCNJ11, CCAH221, ZC3HAV1, FOS, TNFRSF6B, JUN, ATF3, PENK, NR4A3, PDK4, TLR15*
	0042981: regulation of apoptotic process	10	2.36E-04	*CYR61, HNRNPU, TNFRSF6B, SLC30A10, SPRY2, JUN, PDILT, ATF3, NR4A3, PDK4*
	0032879: regulation of localization	16	1.18E-05	*HBEGF, NEXN, PCDH17, KCNJ11, CYR61, SS2, SOCS1, POMC, SLC30A10, SPRY2, HSP70, JUN, CEMIP, NR4A3, TLR15, RRAD*
	0070887: cellular response to chemical stimulus	14	6.85E-05	*HBEGF, CH25H, TIPARP, SOCS1, CCAH221, FOS, TNFRSF6B, SLC30A10, SPRY2, JUN, ATF3, ETV5, NR4A3, PDK4*
	0010033: response to organic substance	14	7.43E-05	*KCNJ11, TIPARP, SOCS1, CCAH221, FOS, TNFRSF6B, SLC30A10, SPRY2, HSP70, JUN, ATF3, NR4A3, PDK4, TLR15*
	0051173: positive regulation of nitrogen compound metabolic process	17	1.72E-05	*HBEGF, EGR4, TXK, CYR61, TIPARP, CCAH221, ZC3HAV1, FOS, SLC30A10, SPRY2, KLF2, HSP70, JUN, ATF3, ETV5, CEMIP, NR4A3*
	0030154: cell differentiation	19	5.89E-06	*NEXN, TXK, CYR61, TIPARP, GRN, SOCS1, FOS, CHAC1, SPRY2, KLF2, HSP70, JUN, PDILT, ATF3, ETV5, NR4A3, NYAP2, NPTX1, MYCL*
	0010604: positive regulation of macromolecule metabolic process	17	2.74E-05	*HBEGF, EGR4, TXK, CYR61, TIPARP, CCAH221, ZC3HAV1, FOS, SLC30A10, SPRY2, KLF2, HSP70, JUN, ATF3, ETV5, CEMIP, NR4A3*
	0031325: positive regulation of cellular metabolic process	16	1.15E-04	*HBEGF, EGR4, TXK, CYR61, CCAH221, ZC3HAV1, FOS, SLC30A10, SPRY2, KLF2, HSP70, JUN, ATF3, ETV5, CEMIP, NR4A3*
	0048731: system development	19	6.46E-05	*HBEGF, NEXN, PCDH17, CYR61, TIPARP, HNRNPU, PAPSS2, FOS, CHAC1, SPRY2, KLF2, JUN, ATF3, NR4A3, HNRNPU, NPTX1, MYCL, NPY5R, ERRFI1*
	0007165: signal transduction	26	4.49E-06	*HBEGF, MCHR2, TXK, CYR61, SS2, TIPARP, SOCS1, LOC422147, CCAH221, RGS1, FOS, TNFRSF6B, POMC, SLC30A10, CHAC1, JUN, ATF3, PENK, NR4A3, NYAP2, GNG4, PDK4, RCAN1, NPY5R, TLR15, RRAD*
Molecular function ontology	0005539: glycosaminoglycan binding	6	2.83E-06	*HBEGF, CYR61, THBS1, CEMIP, SPP1, REG4*
Cellular component ontology	–	–	–	
	0010033: response to organic substance	14	7.43E-05	*KCNJ11, TIPARP, SOCS1, CCAH221, FOS, TNFRSF6B, SLC30A10, SPRY2, HSP70, JUN, ATF3, NR4A3, PDK4, TLR15*
	0051173: positive regulation of nitrogen compound metabolic process	17	1.72E-05	*HBEGF, EGR4, TXK, CYR61, TIPARP, CCAH221, ZC3HAV1, FOS, SLC30A10, SPRY2, KLF2, HSP70, JUN, ATF3, ETV5, CEMIP, NR4A3*
	0030154: cell differentiation	19	5.89E-06	*NEXN, TXK, CYR61, TIPARP, GRN, SOCS1, FOS, CHAC1, SPRY2, KLF2, HSP70, JUN, PDILT, ATF3, ETV5, NR4A3, NYAP2, NPTX1, MYCL*
	0010604: positive regulation of macromolecule metabolic process	17	2.74E-05	*HBEGF, EGR4, TXK, CYR61, TIPARP, CCAH221, ZC3HAV1, FOS, SLC30A10, SPRY2, KLF2, HSP70, JUN, ATF3, ETV5, CEMIP, NR4A3*
	0031325: positive regulation of cellular metabolic process	16	1.15E-04	*HBEGF, EGR4, TXK, CYR61, CCAH221, ZC3HAV1, FOS, SLC30A10, SPRY2, KLF2, HSP70, JUN, ATF3, ETV5, CEMIP, NR4A3*
	0048731: system development	19	6.46E-05	*HBEGF, NEXN, PCDH17, CYR61, TIPARP, HNRNPU, PAPSS2, FOS, CHAC1, SPRY2, KLF2, JUN, ATF3, NR4A3, HNRNPU, NPTX1, MYCL, NPY5R, ERRFI1*
	0007165: signal transduction	26	4.49E-06	*HBEGF, MCHR2, TXK, CYR61, SS2, TIPARP, SOCS1, LOC422147, CCAH221, RGS1, FOS, TNFRSF6B, POMC, SLC30A10, CHAC1, JUN, ATF3, PENK, NR4A3, NYAP2, GNG4, PDK4, RCAN1, NPY5R, TLR15, RRAD*

Besides the GO analysis, DEGs were also plotted to KEGG reference pathways (https://www.kegg.jp/kegg/pathway.html) for down-regulated genes in GNRH1 treated hens compared to AVT hens with FDR < 0.05 (Table 3**)** to collect the molecular interaction, reaction and relation between them. Forty-two KEGG pathways of chicken were assigned. The significantly enriched KEGG pathways (*P* < 0.05) included pathways such as MAPK signalling (8 genes) and Jak-STAT signalling (5 genes).

**Table 3:** KEGG pathway enriched differentially expressed genes for down-regulated genes in GNRH1 treated hens compared to AVT hens with FDR < 0.05

Term	Count	*P*-value	Genes
MAPK signaling pathway	8	7.06E-04	*DUSP1, DUSP4, DUSP5, DUSP6, DUSP8, HSPA2, JUN, MYC*
Jak-STAT signaling pathway	5	9.61E-03	*CISH, IL10RA, SOCS1, SOCS3, MYC*
Cytokine-cytokine receptor interaction	4	9.18E-02	*EDA2R, IL1R2, IL10RA, TNFRSF6B*
ErbB signaling pathway	3	1.10E-01	*HBEGF, MYC, JUN*
Toll-like receptor signaling pathway	3	1.25E-01	*SPP1, JUN, CD80*
Insulin signaling pathway	3	2.09E-01	*HK2, SOCS3, SOCS1*
Influenza A	3	2.44E-01	*SOCS3, HSPA2, JUN*
p53 signaling pathway	2	3.66E-01	*CCNE2, THBS1*
Adipocytokine signaling pathway	2	3.81E-01	*POMC, SOCS3*
Focal adhesion	3	3.98E-01	*THBS1, SPP1, JUN*
ECM-receptor interaction	2	4.21E-01	*THBS1, SPP1*
TGF-beta signaling pathway	2	4.26E-01	*THBS1, MYC*
GnRH signaling pathway	2	4.38E-01	*HBEGF, JUN*

### Confirmation of gene expression differences by RT-qPCR

**Fig. 2:**
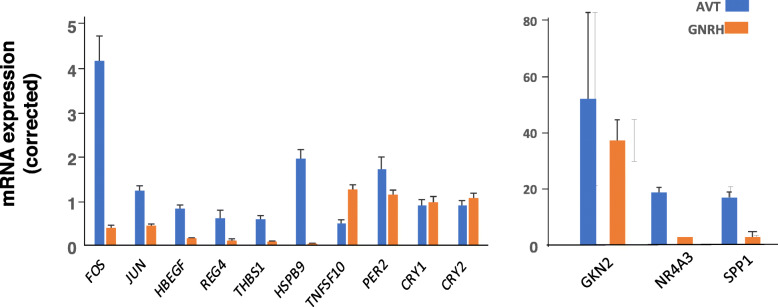
Corrected expression of 13 differentially expressed genes in both AVT (*n* = 8) and GNRH (*n* = 8) treated chicken uterus from RNA-seq analysis measured by RT-qPCR: the genes FOS, *JUN*, *HBEGF*, *REG4*, *THBS1*, *HSPB9*, *TNFSF10*, *PER2, CRY1*, CRY2*,* GKN2, *NR4A3* and *SPP1* are on the Y-axis. Corrected expression on the X-axis was corrected using the geometric mean of *LBR* and *NDUFA1* expression to normalize for any differences between tissues. To better visualize the large differences in expression, the data are presented on two different graphs, the genes in the right-hand panel having greater than 10-fold higher expression than those in the left-hand panel

Differences in expression between GNRH1 and AVT treatments were confirmed for 13 genes (Fig. 2). These included 10 highly DEGs that were both up and down regulated relative to the GNRH1 treatment. In addition, 3 genes (*PER, CRY1* and *CRY2*) that were not indicated to be in the top rank of DEGs but did differ between treatments, were selected for comparison because they were highly expressed and of biological interest in relation to timing events. *PER2* showed differential expression but neither of the *CRY* genes were differentially expressed in the RT-qPCR (Fig. 2). Although the magnitude of changes in gene expression varied greatly, the results of the 13 genes expression correlated to the RPKM values estimated by RNA sequencing.

### Uterus expression of differentially expressed genes at different stages of egg formation

**Fig. 3:**
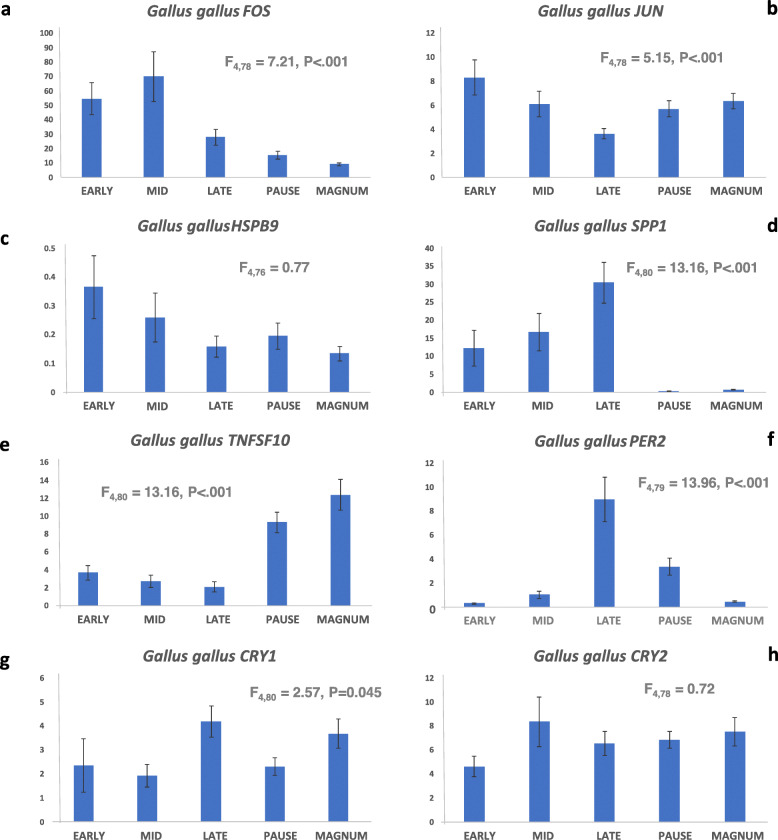
Corrected expression of **a** FOS, **b** JUN, **c** HSPB9, **d** SPP1, **e** TNFSF10, **f** PER2, **g** CRY1 and **h** CRY2 mRNA in uterus tissue at different stages of egg formation measured by RT-qPCR (*n* = 7–11) are represented on the y axis. All genes expression was corrected by the geometric mean of *LBR* and *NDUFA1* expression. The y-axis scale therefore varies depending on the relative level of expression of the genes in the tissue, the larger the number the higher the relative expression of the gene. On the x-axis are the physiological states from which the uterus samples were collected. Early, mid- and late describes the stage of shell formation in the uterus and indicates that the egg was not in the magnum when the samples was taken. Pause represents no egg in the uterus and magnum is when the egg is in that region of the oviduct. The respective F and *P* values from ANOVA are inset in each graph

In order to study further the expression of genes that were apparently regulated between the situations were cuticle production was changed, their expression was measured in the uterus at different stages during egg formation including when there was no egg in the uterus (Fig. 3).

*FOS* expression varied significantly over the egg formation period (F_4,78_ = 7.21, *P* < .001). *FOS* was high during shell formation; the greatest expression was measured at the middle stage of shell formation; the expression was significantly lower when there was no egg in the uterus and the expression at the late period of shell formation was also significantly lower than either mid or early shell formation (Fig. 3a). *JUN* expression varied significantly over the egg formation period (F_4,78_ = 5.15, *P* < .001). The lowest level of expression was measured at the end of shell formation (late) with both the mid and late, pause and white formation expression being significantly lower than the level at the early stage of shell formation (Fig. 3b). *HSPB9* had very low levels of expression during all the egg formation stages and there was no significant difference in expression across the egg formation stages or even when no egg was present (pause) (F_4,76_ = 0.77; Fig. 3c)**.***SPP1* expression increased over the period of shell formation (F_4,77_ = 16.28, *P* < .001), being significantly higher at the end of it. Expression was very low when no egg was present in the uterus (Fig. 3d). *TNFSF10* expression had an opposite trend to *SPP1* (F_4,80_ = 13.16, *P* < .001), with significantly higher expression when no egg was in the uterus but no differences between the stages when an egg was present in it (Fig. 3e). *PER2* varied over the stages categorised (F_4,79_ = 13.96, *P* < .001) with a significant peak of expression at the end of shell formation, whilst the expression was low during the rest of the stages (Fig. 3f). *CRY1* variation was significant (F_4,80_ = 2.57, *P* = 0.045)**,** with a significantly higher expression at the end of shell formation (Fig. 3g). Expression over all the egg formation stages for *CRY2* was similar (F_4,78_ = 0.72; Fig. 3h).

### Correlation of expression between genes during stages of egg shell formation

**Fig. 4:**
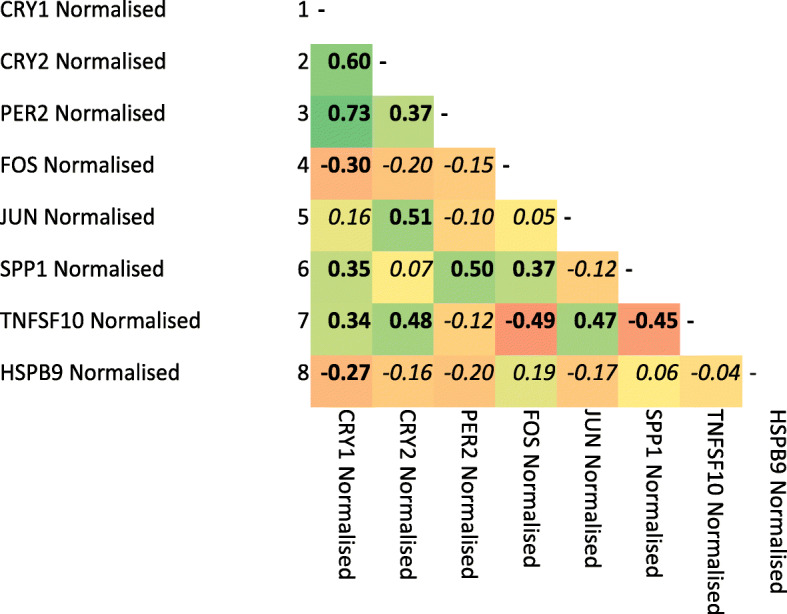
Pearson correlation of uterus gene expression across the different stages of egg shell formation. Text in bold is significantly different from zero. Greener colours indicate positive correlations and redder colours negative correlations and the deeper the colour the stronger the correlation

Significant correlation existed between the expression of genes across the stages of egg formation, with good correlation between *CRY1, CRY2* and *PER2* which show correlations in excess 0.6 (*P* < 0.001; Fig. 4). Other genes showing evidence of correlation around 0.5 (*P* < 0.001) are *TNSF10* with *CRY2*, *FOS*, *JUN* and *SPP1*; *SPP1* with *PER2*; *CRY2* with *JUN* (Fig. 4).

## Discussion

To the best of our knowledge, the present study is the first deep-sequencing analysis of the laying hens’ uterus focused on cuticle deposition, which occurs just before oviposition. The differentially expressed genes identified in this study differ considerably from the results of previous studies that compared when an egg was in the uterus and when there was not [22–24]. We therefore believe that the experimental regimen applied here captures some of the unique events that are important at the very end of the egg forming process including those that result in cuticle deposition.

We present the analysis of the expression profile of genes in the uterus, close to oviposition. There are 32 individuals sampled at this stage of egg formation from 2 experiments, so we have a highly-replicated data set to identify those genes most highly expressed in the uterus at oviposition. Most studies of the transcriptome focus only on the differences between defined states or tissues and only present that data, however there is much information to be gathered from the study of the most abundant genes in a tissue. When the 30 genes with the highest expression in the uterus were annotated as might be expected, several of the genes with extremely high expression at oviposition either encode proteins that are known to be expressed and secreted to form the cuticle [10, 25] or the organic matrix of the eggshell [14] e.g. *MEPE, BPIFB3, RARRES1* and WAP. Other genes are related to mitochondrial oxidative phosphorylation e.g. NADH dehydrogenase subunits, *ND4*, *ND1*, *ND2*, *ND5*, cytochrome c and b related genes and *COX3* that are required for the large amount of energy needed to drive the active secretion of proteins and minerals [26]. These are accompanied by genes important in active transport and energy metabolism, *ACTB*, *GAPDH*, *ATP6* and *ATP1A*. A number of these highly-expressed genes were also differentially expressed in the GNRH1 versus AVT experiment described in this study (Experiment 2); *MEPE*, *COX1*, *COX3*, COX2, *BPIFB3*, Cytochrome b, *ATP6*, *ND5, ATP1A1, ND4, ND2, EIF4A2*, *UBB*, Novel mitochondrial gene, *IGLL1*, *HSPA8*, *RASD1*.

There are however, a number of genes that do not fall immediately into these categories. *IGLL1,* for example, has previously been reported in the uterus in a microarray experiment [27]. This protein was found in the protein component of the deeper layers of the shell [28] and appears to be a major secreted protein responsible for pigeon fanciers lung [29], but other than the strong indication that it is an epithelial secreted protein and its function remains obscure.

Whey acidic protein (ENSGALG00000020972; WAP) was also very abundant and belongs to the first group of genes encoding proteins secreted as part of the egg shell matrix and in particular the cuticle. It appears to be related to genes found in molluscs and may be involved in the termination of mineral accretion on the shell [30]. It seems likely that this gene produces the protein that has been annotated as similar to Kunitz-like protease inhibitor in proteomic studies of the cuticle [10, 25]. It is one of the most abundant proteins in the cuticle [10, 25, 28] and is synonymous with the protein once named as ovocalyxin 25. Ovocalyxin 25 has previously been attributed with antimicrobial properties [31]. However, the possibility that ovocalyxin 25 is critical in termination of mineral deposition prior to cuticle deposition now seems a very strong hypothesis, given similar molecules can change the formation of mollusc shells [30]. The role of matrix proteins in modulating CaCO_3_ crystal formation is well established [32], but both proposed roles are not mutually exclusive.

Overall, the most abundant genes in the uterus reported here are broadly in agreement with the 5 top expressed genes quoted in a recent NGS study that also featured the uterus with the unidentified transcript ENSGALG00000044239 likely being homologous to ENSGALG00000018361, NADH-ubiquinone oxidoreductase chain 5 in this study. The absence of whey acidic protein (ENSGALG00000020972) in that study, which was the 3rd highest in the list reported here, may reflect the time of sampling in this study which was later in shell formation and would reinforce the role of this gene at the end of calcification and in cuticle formation.

Given the focus of the studies on the cuticle, we were also interested in genes that were highly expressed and that might be related to the timely secretion of the cuticle just before oviposition [6]. A notable gene of interest was ENSGALG00000005521, which encodes Period Circadian Regulator 2 (*PER2*) with an expression of 10.1 log cpm. There were also high levels of other components of the circadian clock mechanism, Cryptochrome Circadian Regulator 2 (*CRY2*) and Cryptochrome Circadian Regulator 1 (*CRY1*), at 9.4 log cpm and 7.6 log cpm respectively. We also found *CLOCK* (3.9 log cpm) and the gene encoding BMAL1, (*ARNTL*, 5.5 log cpm) at what were average levels of expression for this study. The presence of a functioning clock [33] in peripheral tissues is not new [34] and the elements of the clock have been observed before in the chicken uterus where they were seen to cycle [35]. *PER2* was also differentially expressed in a NGS study comparing hen uteri 15 h apart [24]. Although not in the list of genes with the most significant differences in expression, we also found significant differences between the AVT and GNRH1 treated hens in the expression of *PER2* (*P* = 0.0028), *CRY2* (ENSGALG00000008436; *P* = 0.0001), *CRY1* (ENSGALG00000012638; *P* = 0.0002), *CLOCK* (ENSGALG00000013793; *P* = 0.001) and *ARNTL* (ENSGALG00000005378; *P* = 0.00002). These differences combined with the high level of expression of *PER* and *CRY* genes, confirmed by RT-qPCR, suggests that Clock genes may be important in driving the cyclic expression of genes in the uterus. When examined over the egg laying cycle the expression of *PER2* showed strong cyclicity related to the time in the egg formation cycle. Although not as distinct a pattern, *CRY1* but not *CRY2*, expression was also highest at the end of egg formation. Cyclicity of the clock related genes has previously been observed in the oviduct of mice which could be entrained by external stimuli including endocrine signals [36]. In that study clock time appears to have been shifted by signals that alter the timing of the ovulatory surge [36]. Such a phase shift could be critical in the timing of cuticle deposition in the hen’s uterus or other key events during shell formation and therefore provides a testable hypothesis.

A further aspect of this study was to compare the uterus transcriptome from the extremes of the population distribution for the trait. A photograph of what the extremes of the cuticle deposition distribution look like is available in the supplemental material of a recent paper along with the associated the genetic parameters of the trait [18]. Hens at the low end of the distribution have virtually no cuticle whilst those at the top have a strong even cuticle deposition resulting in a large numerical difference in cuticle reflectance between the eggs laid by the hens that were used for transcriptome analysis. Interestingly we did not find any detectable differences in gene expression between these two groups. One explanation could be that the genetic effects that result in large phenotypic differences in this trait may not be expressed in the uterus, but may reside in neural structures that control release of the cuticle proteins in the uterus or in a timing mechanism related to ovarian-uterus interaction or other unknown mechanisms. Yet we found no evidence for the AVT prostaglandin system that is thought to mediate the brain to ovary signalling of oviposition timing being involved in cuticle deposition [6].

Alternative hypothesis would be that undetectable differences in expression might be responsible for major changes in cuticle deposition. Given that in the endocrinological intervention using AVT/GNRH we observed clear effects on expression, it seems likely that if there were even small changes in expression we would have detected these. Both experiments had the same statistical power. Given the prominence of clock genes in the AVT/GNRH experiment the possibility that variation in their protein expression rather than gene expression is a possibility, indeed in many clock mechanisms there are observations of differences in the profile of the different components of the clock [37]. These differences might be genetic differences in, for example, the half-life of clock proteins that would not be apparent in the transcriptome [37].

In experiment 2 we compared gene expression in birds where we had manipulated the hypothalamo-pituitary-gonadal-oviduct axis pharmacologically using either GNRH1 or AVT. Birds on each treatment laid eggs at the same time but either with (GNRH1) or without (AVT) a cuticle. The most differentially expressed genes in this case fell into the GO molecular function glycosaminoglycan binding (0005539): *HBEGF*, *CYR61*, *THBS1*, *CEMIP*, *SPP1*, *REG4*. For those genes tested, all were confirmed to have a lower level of expression in the GNRH1 group who had completed a normal albeit advanced egg formation cycle than the AVT group by RT-qPCR. In experiment 3 where we examined gene expression at different stages of the egg production cycle these same genes expression varied quite significantly. For example *SPP1*, widely known as osteopontin in relation to mineralisation, was clearly higher at the end of the shell formation period, a fact noted previously [38] and recently in a transcriptome study [22]. These genes must presumably reduce their expression rapidly after oviposition which would make sense as there is no longer an egg in the oviduct and no requirement for events associated with glycosylation of matrix or cuticle proteins.

A further category of genes identified from experiment 2 was a group of genes that had both relatively high expression and a large significant difference in expression, where expression in the GNRH1 group was lower than in the AVT group. This category included the immediate early genes *JUN* and *FOS* as well as *NR4A3*, and *HSPB9*. There were also several genes associated with the KEGG pathways for both MAPK and Jak-STAT signalling pathways. The immediate early gene products, JUN and FOS, classically act as transcription factors and can form dimers to activate transcription [39]. As the name suggests these genes respond rapidly to stimuli which could explain why the AVT group expression was much higher uterus responded to the AVT injection. This seems plausible because in the natural cycle (experiment 3) expression was lower in the late phase of shell formation. In the chick oviduct JUN and FOS have also been shown to be responsive to the steroid environment, increasing with oestrogen and decreasing in response to progesterone [40–42]. In birds the secretion of progesterone peaks at ovulation and is critical to maintaining the ovulatory surge [43]. Previously, it had been proposed that progesterone might be important to cuticle formation [44]. However, in a previous publication we utilised the lack of a progesterone on a pause day to demonstrate there was no direct effect of the presence or absence of a progesterone ovulatory surge on cuticle deposition [6] although arguably the timing effect of the previous surge could still be critical.

Two genes had both high levels of expression and a large significant difference between the AVT and GNRH group. For these genes, TNFSF10 and GKN2 (Ovocalyxin 21), expression was lower in the AVT group which produced eggs without cuticles versus the GNRH group that did have cuticles on the eggs produced. However, it should be noted, that the differences in GKN2 expression was not validated by qPCR. Tumor Necrosis Factor Superfamily Member 10, *TNFRSF10* is known as TRAIL and is the ligand for receptors known as TRAILR, but can also be bound by the decoy receptor TNFRSF11B, also known as osteoprotegerin (OPG). OPG is present in the uterus and is highly upregulated as the steroid environment changes with sexual maturity [45]. OPG, under the influence of oestrogen, is thought to prevent the activation of death domain receptors within the oviduct [46] by TRAIL related apoptosis. In the current study, *TNFRSF10* had higher expression in the GNRH1 group, which may be related to subsequent fluctuation in ovarian steroids which maintain the oviduct and the extremely rapid regression and apoptosis which occurs when steroid support is removed. This observation was confirmed by a transcriptome study comparing the uterus during and before egg shell calcification where *TNFSF10 was higher when no egg was in the uterus* [22]*.*

Although relatively few of the top differentially expressed genes identified in this paper are in common with the study of Kahn [22], those that are common to both studies change very dynamically during the egg formation cycle. These include *BPIFB3* (ovocalyxin 36), *BPIL3*, *GAL3ST2*, *GNG4*, *HABP2*, *NPTX1*, *POMC*, *SLC13A5*, *SPP1*, *TNFRSF6B* and *TNFSF10*. Using a microarray approach, Brionne [34] profiled the expression of genes in the uterus in the presence of a hard shelled egg and identified a number of genes some of which are common to both studies; *BPIFB3* (ovocalyxin 36), *GKN2, BPIL3*, *POMC*, *PENK*, *SPP1* and *TNFRSF6B* [23] whilst in a similar transcriptome study using RNA sequencing there was shared differential expression observed in the uterus with and without an egg of *BPIL3, DUSP4, GAL3ST2, GKN2, MXD4, PENK, RCAN1* and again *POMC* and *SPP1* [24]. The genes in common between our study and these studies are more likely to be involved in shell formation and therefore those genes which are not in common, such as the clock genes and the immediate early genes as prime candidates for the control of cuticle deposition.

## Conclusions

By manipulating the production of eggs with or without cuticle endocrinologically we have demonstrated two categories of genes that may be responsible for controlling the production of the cuticle. Clock genes, which determine the timing of cellular events and ensure that the cells respond appropriately to their environment, are possible mediators of cuticle deposition as the timing of deposition is very tightly controlled. Clock gene expression during shell formation show dynamic changes that support the hypothesis. Immediate early genes, which are critical in the activation of cellular processes by external factors, have large fold differences between the physiological states where cuticle has been secreted and those where it has not. The lack of differentially expressed genes when the top and tail of the cuticle deposition were compared suggests that the uterus itself may not be the site of genetic differences in cuticle deposition, therefore other tissues may be responsible for genetic variability of this trait.

## Methods

### Animals and sampling details

All hens used in experiments were sourced commercially as day old layer hen chicks Lohmann Brown (Lohmann GB, Worcester, England) and were housed in our facilities at Roslin. Hens from the top and tail of the distribution were collected from a Lohmann Tierzucht flock with permission. In total 58 animals were used to provide material for the studies in this paper and material from a further 36 animals which had been collected for at the same time as material utilised in previous publications [47].

Hens were reared and maintained as described previously; for experiment 1, the study investigating the Rhode Island Red hens that laid eggs from the top and tail of the distribution i.e. good or poor cuticle, were as described in Bain et al. [10]. This is a commercial pure line that is used in the breeding of Lohmann brown hens and meant we had around 2000 70-week-old hens to select from.

Hens were reared in cages in an environmentally controlled poultry house, receiving 16 h of light per day and were fed following standard commercial practice. Hens were selected based on measurements made at 31 and 50 weeks of age [18]. Genetic correlations across age for the trait of cuticle deposition was near 1 [18] suggesting hens that were laying good or poor cuticle covered eggs earlier in life would continue to do so and indeed that was the case. Nonetheless, two eggs from each hen were measured to ensure the hens were still laying eggs with good or poor cuticle. Twenty-Five High and 17 Low individuals from the distribution were available with tissue and eight of the most extreme individuals were selected from each group for RNA-Seq analysis. Hens were killed immediately after oviposition as specified in Schedule 1 of the UK Animals (Scientific Procedures) Act (1986) using an intra-venous injection of 200 mg/kg of Pentobarbitone Sodium (Euthatal, Merial Animal Health Limited, Harlow, Essex, UK).

For experiment 2, the studies utilizing treatments to create the presence or absence of cuticle were as detailed in Wilson et al. [6]. Briefly the Lohmann Brown (Lohmann GB, Worcester, England) layer hens (*Gallus gallus domesticus*) were maintained on a 28 h cycle to synchronise their ovulatory cycle.

Uterine tissue was collected from hens that laid eggs with (*n* = 8) or without (*n* = 8) cuticle at the same time. This was achieved by causing premature oviposition on a 28-h daily cycle with either GNRH1 peptide where the cuticle was deposited as normal or by using AVT where eggs are produced with no cuticle. This approach and all the peptides used were exactly as used and validated previously to study the physiology of cuticle deposition [6]. Briefly, GNRH1 (28.9 × 10^− 6^ mol/kg body weight) was administered intravenously 10 h before a normal oviposition on the 28-h ahemeral light cycle and intravenously administered arginine vasotocin (AVT) (1.05 × 10^− 6^ mol/kg body weight) 4.5 h prematurely. Hens were reared on the floor, to peak-of-lay (22–28 weeks), following commercial management practice, except for the lighting, which was 14 L:10D. Hens were transferred to individual cages prior to the experimental period on a 28-h ahemeral light/dark cycle (14 L: 14D).

Hens were killed immediately after oviposition as specified in Schedule 1 of the UK Animals (Scientific Procedures) Act (1986) using an intra-venous injection of 200 mg/kg of Pentobarbitone Sodium (Euthatal, Merial Animal Health Limited, Harlow, Essex, UK).

For experiment 3 uterus samples were collected at the same time as the magnum samples featured in Gong et al. [47], this allowed uterus samples across the entire spectrum of 24 h egg formation cycle to be obtained and the stage of egg formation was determined by microscopical examination of the shell. Briefly, uterus tissue was collected either when the egg was in the magnum where the albumen is secreted (*n* = 7), in the uterus (shell gland) and recorded as early (*n* = 9), mid- (*n* = 9), and late (*n* = 9) calcification based on electron microscopy of the shell or during a pause day (*n* = 11) when there was no ovulation and therefore, no egg in the oviduct.

Selection of sample size was based on previous studies on cuticle deposition [6] to supply data for variance and effect size using for a power calculation (Genstat, 18th edition, VSN International Ltd., Hemel Hempstead, England). The number of animals was the minimum number required to get the a statistically significant effect on cuticle deposition with a power of 1.

Animals in experiment 1 were assigned to experimental treatments by ranking and randomization on body weight. The individual cage was the experimental unit in all experiments.

### Zootechnical data, tissue collection and storage

Hens were weighed at cull and the whole oviduct removed and weighed, minus any egg it contained. The uterus pouch was laid out with the pouch facing away from the dissector. A small incision was made in the centre of the pouch and the uterine fluid removed. A longer incision (~ 5 mm) was then made through the pouch away from the dissector and an approximately square section through the pouch was taken (< 100 mg). The uterine tissues in experiment 1–3 were collected into RNAlater (Life Technologies, Paisley, Scotland), stored overnight at 4 °C and subsequently at − 80 °C prior to total RNA extraction.

### Measurements of egg cuticle deposition and shell pigmentation

Cuticle and pigment were measured spectrophotometrically as described previously [6, 10]. Briefly, absorbance of the eggshell at 640 nm was measured before and after staining with a cuticle-specific dye consisting of tartrazine/lissamine green B (Sigma-Aldrich, Poole, Dorset, England). Pre-measurement; absorbance at 640 nm on the unstained egg (Abs @ 640 nm) is reflects the intensity of the pigmentation of the egg, post measurement; difference in absorbance at 640 nm between the unstained egg and the same egg stained with cuticle dye (ΔAbs @640 nm). This represents the amount of cuticle deposition.

### RNA isolation, sequencing and quality control analysis

RNA was isolated from each tissue sample for sequencing using TRIzol (Invitrogen Ltd., Renfrewshire, Scotland) as described previously. RNA was treated with DNAse I and purified using a RNeasy Mini Kit (Qiagen, Manchester, England). The quality and concentration of RNA was checked with a Nanodrop ND-1000 Spectrophotometer (Thermo Scientific; Waltham, MA USA). 2-4μg of total RNA from each sample were used to make individual bar-coded libraries using Illumina TruSeq RNA Sample Preparation kit (Illumina, Cambridgeshire, England). These libraries were sequenced on an Illumina HiSeq 2000 sequencer (HiSeq high output v4 50PE; Illumina, San Diego, CA, USA). raw data was quality controlled using the FastQC package (Babraham bioinformatics, Cambridgeshire, England). Adapters were trimmed from reads using cutadapt version 1.3 with the parameters -q 30 -m 50 -a AGATCGGAAGAGC.

### Transcriptomic data analysis

Trimmed reads were aligned to the *Gallus gallus* reference genome (Ensembl v82, Galgal 4) with the *TopHat2 Aligner* (http://ccb.jhu.edu/software/tophat; version 2.0.13) using default parameters [48]. Expression levels of the transcripts were quantified as RPKM (reads per kilobase per million). Read counts were generated using *HTSeq-count* (version 0.6.0) [49] and *Python* (version 2.7.3) by Edinburgh Genomics, the University of Edinburgh. Subsequent data filtering and analysis was performed with *EdgeR* software [50, 51]. Genes with RPKM < 1 in at least two samples were not included in the analysis. After normalization, gene-wise generalized linear models (GLM) were fitted on the samples followed by likelihood ratio test to determine differentially expressed genes (DEGs) with a *P*-value of 0.05 as a threshold. Ensembl gene IDs from each group with a fold-change > 1.5 or < − 1.5 were uploaded to the DAVID Functional Annotation Tool [52, 53] and analysed for gene ontology (GO) and Kyoto Encyclopaedia of Genes and Genomes (KEGG) enrichment.

### RT-qPCR

mRNA expression differences were verified by qPCR using cDNA from 8 uterine tissue samples from the two treatments (experiment 2). For the tissue expression experiment, cDNA from between 7 and 11 uterus samples was measured for each egg formation stage. Primers were designed using Primer 3 software [54] on exon-exon spans when possible for some of the differentially expressed genes (Additional file 6: Table S6). The qPCR was performed using the Agilent Brilliant III Ultra-Fast SYBR Green qPCR Mix and MX3005P real time system (Agilent; Santa Clara, CA USA). The measurements were carried out in duplicate, essentially as described previously [45, 55]. To confirm that only products of the correct length, free from primer dimers, were amplified by each primer pair; the products were assessed by electrophoresis and sequenced directly to confirm identity.

Concentrations were normalized using a geometric mean of the expression of two ‘housekeeping genes’, *LBR* and *NDUFA1* [56]. One-way ANOVA was used for statistical analysis of data using Minitab Express V.1.3.0. In experiment 3, the Pearson correlation matrix for gene expression was produced using GenStat 18th edition (VSN International Ltd., Hemel Hempstead, England).

Conference proceedings; An brief account of this work has been presented at the UK branch meeting of the Worlds Poultry Science Association [57].

## Supplementary information

**Additional file 1 Table S1**. Dataset of 12,253 genes with RPKM > 1 resulting from the comparison of the top and tail of the cuticle deposition distribution.

**Additional file 2 Table S2**. Dataset of 12,248 genes with RPKM > 1 from two experimental manipulations of oviposition timing through the injection of GNRH1 and AVT.

**Additional file 3 Table S3**. DEGs in GNRH1 treated hens uterus compared to AVT treated hens uterus.

**Additional file 4 Table S4**. Significantly up-regulated genes with a fold-change > 1.5 in GNRH1 compared to AVT treated hens (i.e. with versus without cuticle).

**Additional file 5 Table S5**. Significantly down -regulated genes with a fold-change < − 1.5 in GNRH1 compared to AVT treated hens (i.e. with versus without cuticle).

**Additional file 6 Table S6**. Gene names, abbreviations and primers used in RT-qPCR.

## Data Availability

The data sets supporting the results of this article are publicly available in the European Nucleotide Archive (ENA) repository under the accession numbers PRJEB32554 (https://www.ebi.ac.uk/ena/browser/view/PRJEB32554) and PRJEB32460 (https://www.ebi.ac.uk/ena/browser/view/PRJEB32460). Trimmed reads were aligned to the *Gallus gallus* reference genome (Ensembl v82, Galgal 4) Accession number PRJNA13342 (https://www.ncbi.nlm.nih.gov/assembly/GCF_000002315.3/).

## Abbreviations

Abs@640 nm: Absorbance of 640 nm wavelength light by the egg before staining, this represents egg colour
AP-1: Transcription factor subunit
*ARNTL*: Aryl hydrocarbon receptor nuclear translocator like, also known as BMAL1
*ATP1A1*: ATPase Na+/K+ transporting subunit alpha 1
*ATP6*: ATP synthase F0 subunit 6
AVT: Arginine vasotocin
BPIFB3(OVX-36): BPI fold containing family B member 3 (Ovocalyxin 36)
*BPIL3*: Bactericidal/permeability-increasing protein-like 3
*CEMIP*: Cell migration inducing hyaluronidase 1
*CLOCK*: Clock circadian regulator
CLU: Clusterin
*COX1*: Cytochrome c oxidase subunit I
COX2: Cytochrome c oxidase subunit II
*COX3*: Cytochrome c oxidase subunit III
*CRY1*: Cryptochrome circadian clock 1
*CRY2*: Cryptochrome circadian clock 2
*CYR61*: Cysteine rich angiogenic inducer 61
DEGs: Differentially expressed genes
*DUSP4*: Dual specificity phosphatase 4
*EIF4A2*: Eukaryotic translation initiation factor 4A2
FDR: False discovery rate
*FOS*: Proto-oncogene c-FOS
*GAL3ST2*: Galactose-3-O-sulfotransferase 2
*GKN2*: Gastrokine 2 (Ovocalyxin 21)
*GNG4*: *G protein subunit gamma 4*
GNRH1: Gonadotropin-releasing hormone 1
GO: Gene Ontology
*HABP2*: *Hyaluronan binding protein 2*
*HBEGF*: Proheparin-binding EGF-like growth factor
*HSPA8*: Heat shock 70 kDa protein 8
*HSPB9*: Heat shock protein family B (small) member 9
*IGLL1*: *Immunoglobulin lambda-like polypeptide 1*
*JUN*: Jun proto-oncogene
KEGG: Kyoto Encyclopaedia of Genes and Genomes
*LBR*: Lamin B receptor
logCPM: Log counts per million
MAPK: Mitogen-activated protein kinase
MEPE (OC-116): Matrix extracellular phosphoglycoprotein (ovocleidin-116)
*MXD4*: MAX dimerization protein 4
*ND1*: NADH dehydrogenase subunit 1
*ND2*: NADH dehydrogenase subunit 2
*ND4*: NADH dehydrogenase subunit 4
*ND5*: NADH dehydrogenase subunit 5
*NDUFA1*: NADH:ubiquinone oxidoreductase subunit A1
*NPTX1*: *Neuronal pentraxin 1*
*NR4A3*: Nuclear receptor subfamily 4 group A member 3
*PENK*: Proenkephalin
*PER2*: Period circadian protein homolog 2
*POMC*: *Proopiomelanocortin*
RARRES1 (OVX-32): Retinoic acid receptor responder 1 (Ovocalyxin 32)
*RASD1*: *Ras related dexamethasone induced 1*
*RCAN1*: Regulator of calcineurin 1
*REG4*: Regenerating family member 4
RNA-Seq: RNA sequencing
RPKM: Reads per kilobase per million
RT-qPCR: Reverse transcription quantitative chain polymerase reaction
*SLC13A5*: Solute carrier family 13 member 5
*SPP1*: Secreted phosphoprotein 1 (also known as Osteopontin)
*THBS1*: Thrombospondin 1
*TNFRSF6B*: TNF receptor superfamily member 6b
TNFRSF11B: TNF receptor superfamily member 6b
*TNFSF10*: Tumor Necrosis Factor superfamily member 10
TRAIL: Tumor Necrosis Factor superfamily member 10
*UBB*: Ubiquitin B
WAP (OVX-25): Whey acidic protein (Ovocalyxin 25)
ΔAbs@640 nm: The difference in absorbance of 640 nm wavelength light by the egg before and after staining, this represents the cuticle
